# A critical role of Oct4A in mediating metastasis and disease-free survival in a mouse model of ovarian cancer

**DOI:** 10.1186/s12943-015-0417-y

**Published:** 2015-08-11

**Authors:** Chantel Samardzija, Rodney B Luwor, Mila Volchek, Michael A Quinn, Jock K Findlay, Nuzhat Ahmed

**Affiliations:** Women’s Cancer Research Centre, Royal Women’s Hospital, Victoria, 3052 Australia; Department of Obstetrics and Gynaecology, University of Melbourne, Victoria, 3052 Australia; Department of Surgery, University of Melbourne, Royal Melbourne Hospital, Victoria, 3052 Australia; Department of Anatomical Pathology, Royal Women’s Hospital, Victoria, 3052 Australia; Hudson Institute of Medical Research, Victoria, 3168 Australia; Fiona Elsey Cancer Research Institute, Ballarat, Victoria, 3353 Australia; Fiona Elsey Cancer Research Institute, 106-110 Lydiard Street South, Central Park, Ballarat, Victoria, 3363 Australia

**Keywords:** Ovarian carcinoma, Cancer stem cells, Metastasis, Ascites, Chemoresistance, Recurrence, Oct4A

## Abstract

**Background:**

High grade epithelial ovarian cancer (EOC) is commonly characterised by widespread peritoneal dissemination and ascites. Metastatic EOC tumour cells can attach directly to neighbouring organs or alternatively, maintain long term tumourigenicity and chemoresistance by forming cellular aggregates (spheroids). Cancer stem-like cells are proposed to facilitate this mechanism. This study aimed to investigate the role of Oct4A, an embryonic stem cell factor and known master regulator of pluripotency in EOC progression, metastasis and chemoresistance.

**Methods:**

To investigate the expression of Oct4A in primary EOC tumours, IHC and qRT-PCR analyses were used. The expression of Oct4A in chemonaive and recurrent EOC patient ascites-derived tumour cells samples was investigated by qRT-PCR. The functional role of Oct4A in EOC was evaluated by generating stable knockdown Oct4A clones in the established EOC cell line HEY using shRNA-mediated silencing technology. Cellular proliferation, spheroid forming ability, migration and chemosensitivty following loss of Oct4A in HEY cells was measured by *in vitro* functional assays. These observations were further validated in an *in vivo* mouse model using intraperitoneal (IP) injection of established Oct4A KD clones into Balb/c nu/nu mice.

**Results:**

We demonstrate that, compared to normal ovaries Oct4A expression significantly increases with tumour dedifferentiation. Oct4A expression was also significantly high in the ascites-derived tumour cells of recurrent EOC patients compared to chemonaive patients. Silencing of Oct4A in HEY cells resulted in decreased cellular proliferation, migration, spheroid formation and increased chemosensitivity to cisplatin *in vitro*. IP injection of Oct4A knockdown cells *in vivo* produced significantly reduced tumour burden, tumour size and invasiveness in mice, which overall resulted in significantly increased mouse survival rates compared to mice injected with control cells.

**Conclusions:**

This data highlights a crucial role for Oct4A in the progression and metastasis of EOC. Targeting Oct4A may prove to be an effective strategy in the treatment and management of epithelial ovarian tumours.

**Electronic supplementary material:**

The online version of this article (doi:10.1186/s12943-015-0417-y) contains supplementary material, which is available to authorized users.

## Introduction

Epithelial ovarian cancer (EOC) remains the most prevalent of all the gynaecological malignancies with approximately 250,000 women diagnosed worldwide with the disease each year [1]. Despite extensive treatment regimens including cytoreductive surgery and chemotherapy, the disease carries a poor prognosis with a 5 year mortality rate greater than 70 % [2]. This is predominately due to exceptionally high rates of disease recurrences driven by widespread peritoneal metastasis and ascites [3]. Although the metastasis of EOC is a highly complex and multi-stage process [4], it is further emphasised by the lack of anatomical barriers within the pelvic cavity. Consequently, malignant tumour cells from the primary ovarian site are freely able to exfoliate directly into the peritoneal cavity. Here they directly attach to the mesothelial lining of the peritoneum before invading adjacent pelvic organs including the bowels, bladder and liver. Alternatively, exfoliated tumour cells can also maintain long term tumourigenicity within the peritoneal cavity by forming non-adherent multicellular tumour aggregates (spheroids) in the ascites microenvironment [5]. This combined with the emergence of drug resistant tumour cells following extensive chemotherapy regimens presents a major challenge in the management of the disease [6, 7]. In order to reduce recurrent EOC tumour burden and improve overall patient survival rates, the molecular mechanisms involved in ascites-mediated EOC progression must be investigated.

Recent evidence suggests solid tumours including those of ovarian origin contain a sub-population of tumour cells exhibiting unlimited self-renewal and pluripotent abilities [8]. These cells, termed cancer stem cells (CSCs) have gained considerable momentum in cancer biology over the past decade and are hypothesized to mediate tumour cell survival [9], sustain cytotoxic pressure [10, 11] and initiate metastatic recurrence [12]. Indeed, we and others have shown recurrent chemoresistant ovarian tumour cells to be enriched in CSC-like cells and stem cell pathway mediators, indicating that CSCs may contribute to the progression of disease [13–17]. Overall, this suggests that targeting CSCs may provide an effective and accurate method of treating recurrent EOC disease [18–20]. It is therefore vital that tumour specific CSCs as well as the pathways regulating their survival are identified and explored.

Oct4 (Oct3/4 or POU5F1), an embryonic stem cell factor and member of the POU family of transcription factors, has been extensively studied in solid tumours including those of the lung [21], brain [22], and breast [23] where it has closely been related to tumour progression [24], self-renewal [25] and drug resistance [26]. In developmental biology, it is known to play a pivotal role for the maintenance of totipotency in primary blastomeres and pluripotency of the inner cell mass of developing mammalian embryos [27, 28]. While up regulation of Oct4 sustains an undifferentiated pluripotent stem cell state, a loss of Oct4 induces stem cells to undergo differentiation, producing a heterogeneous population of highly specialized daughter cells [29]. Unfortunately, several studies previously investigating Oct4 in relation to tumour progression and chemoresistance have failed to recognize the several Oct4 isoforms generated by alternative splicing [30] termed Oct4A, Oct4B and Oct4B1. Of these isoforms, only the nuclear-specific Oct4A isoform is known to be capable of regulating the pluripotent nature of stem cells [31, 32]. This along with the several known pseudogenes [33], makes investigating and interpreting the current literature on Oct4 complex. Hence, to elucidate the expression and the biological functions of Oct4 in the context of cancer stem cells, it is important to discriminate between the several isoforms and pseudogenes of Oct4.

In this study, for the first time using Oct4A specific antibody and primers we determined the expression of Oct4A in a range of serous ovarian tumours of different histological grades, and in tumour cells isolated from the ascites of recurrent and chemonaive EOC patients. Our results reveal that the expression of Oct4A significantly correlated to serous ovarian tumour dedifferentiation and was significantly elevated in the isolated tumour cells of chemotherapy treated recurrent patients compared to untreated chemonaive patients. Using a highly aggressive and metastatic ovarian cancer cell line (HEY) which sustains high endogenous expression of Oct4A, we show that suppression of Oct4A resulted in the loss of CSC-associated expression of Lin28, Sox-2, EpCAM and CD44. This was associated with a decrease in cellular proliferation, migration, spheroid forming abilities and resulted in enhanced sensitivity to cisplatin *in vitro*. These results correlated with those obtained from *in vivo* mouse xenograft studies. Mice transplanted with Oct4A knockdown cells demonstrated significantly reduced tumour burden and abrogation of tumour invasive ability, which overall resulted in significantly increased survival rates compared to mice injected with vector control cells. These data emphasize the need to explore further the effect of Oct4A expression in pre-clinical ovarian cancer models.

## Results

### Oct4A is over expressed in primary serous ovarian carcinomas and in the ascites-derived isolated tumour cells of recurrent patients

**Fig. 1:**
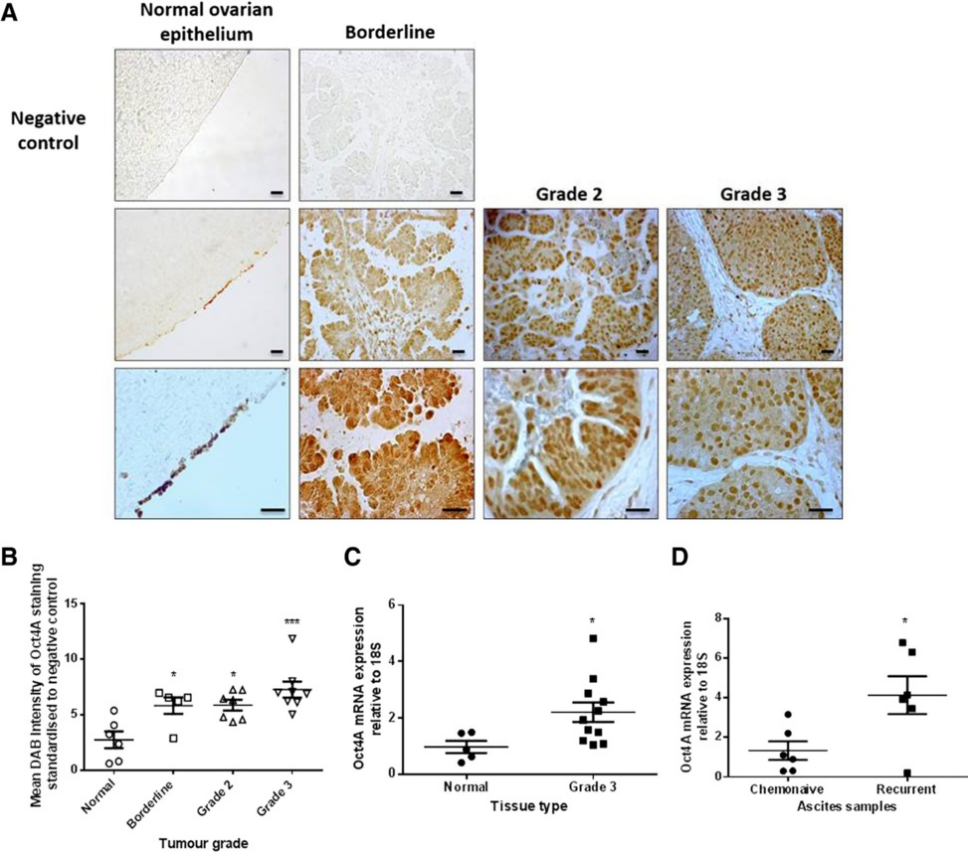
Expression and localization of Oct4A in primary serous epithelial ovarian tumours. **a** Representative immunohistochemical staining of Oct4A in normal (*n* = 7), borderline (*n* = 5), grade 2 (*n* = 7) and grade 3 (*n* = 8) primary ovarian tumours. Positive Oct4A expression is indicated by intense nuclear staining. Images are set at 200x (top panels including negative controls) and 400x (bottom panels). Scale bars represent 10 μM. **b** Quantification of Oct4A staining using Image J software which recognizes total DAB intensity. Variations in staining were determined by subtracting the negative control DAB reading from the Oct4A DAB reading for each tissue sample. Data is presented as the mean ± SEM of Oct4A DAB staining intensity. Significance is indicated by *P < 0.05, **P < 0.01 or ***P < 0.001 as determined by One-way ANOVA using Dunnett’s Multiple Comparison post-test compared to normal ovarian epithelium tissue. **c** Oct4A mRNA expression in primary normal ovarian epithelium tissues (*n* = 6) and primary Grade 3 serous epithelial ovarian tumours (*n* = 11) was determined by qPCR analysis. Relative quantification of Oct4A mRNA expression is standardized to 18S housekeeping gene and normalized to normal ovarian epithelium tissues. Data is expressed as the mean ± SEM of log_2_ transformed data of samples performed in triplicate. Significant increase of Oct4A expression in Grade 3 serous EOC tumours is indicated by *P < 0.05 as determined by student's t-test. **d** qPCR analysis of Oct4A expression in tumour cells isolated from the ascites of chemonaive (*n* = 6) and recurrent (*n* = 6) serous EOC patients. Relative quantification of Oct4A mRNA expression was standardized to 18S housekeeping gene. Data is expressed as the mean ± SEM of Oct4A mRNA expression of each sample performed in triplicate. Significance is indicated by *P < 0.05 as determined by student's t-test

To first establish whether Oct4A is expressed in primary serous ovarian tumours, a total of 26 paraffin embedded cases (Table 1), consisting of 6 normal ovarian epithelia, 5 well differentiated borderline serous tumours, 7 moderately differentiated grade 2 serous tumours, and 8 poorly differentiated grade 3 serous tumours were analysed by immunohistochemistry using a human Oct4A-specific antibody specifically targeting the N-terminal of the Oct4 protein. Enhanced expression of Oct4A was observed in ovarian tumours compared to normal ovarian epithelium samples (Fig. 1a & Additional file 1: Figure S1). This expression was noted in both the cytoplasm and nuclei of tumour cells, with a greater number of nuclear staining observed in grade 2 and grade 3 tumours compared to normal and borderline specimens. However, a small section of ovarian surface epithelium stained positive for Oct4A. It is not certain whether this is true Oct4A staining or simply an ‘edging effect’. A significant difference in Oct4A staining (both cytoplasmic and nuclear) was however observed between all serous tumour samples and normal ovarian tissues (Fig. 1b) with weak Oct4A staining observed in normal ovarian epithelium tissue samples (DAB reading: 2.75 ± 0.76), moderate staining in borderline (5.83 ± 0.75) and grade 2 (5.9 ± 0.48) tumours and moderate to high in and grade 3 tumours (7.28 ± 0.72). Real-time PCR analysis using a primer set specifically targeting exon 1 of the Oct4 gene also confirmed significantly increased expression of Oct4A at the mRNA level with 50 % of poorly differentiated grade 3 serous tumour samples exhibiting moderate to high expression of Oct4A compared to normal ovarian samples (Fig. 1c) (Table 2).

**Table 1:** Description of patient samples used for IHC analysis

Sample no.	Diagnosis	FIGO stage	Age	Pre-operative CA125	Survival
Normal					
1	Normal ovarian epithelium	-	NA	-	-
2	Normal ovarian epithelium	-	61	-	-
3	Normal ovarian epithelium	-	69	-	-
4	Normal ovarian epithelium	-	65	-	-
5	Normal ovarian epithelium	-	62	-	-
6	Normal ovarian epithelium	-	NA	-	-
Borderline					
1	Serous cystadenocarcinoma	Ib	50	12	4 years 10 months ALC
2	Serous cystadenocarcinoma	Ic	31	117	4 years 9 months^a^
3	Serous cystadenoma	Ia	60	1237	5 years ALC
4	Serous cystadenoma	Ic	32	34	4 years 8 months^a^
5	Papillary serous cystadenocarcinoma	Ia	47	NA	5 years 8 months ALC
Grade 2					
1	Papillary serous cystadenocarcinoma	IIIc	71	NA	8 months ALC
2	Papillary serous cystadenocarcinoma	IIIc	43	428	3 years 7 months until death
3	Papillary serous cystadenocarcinoma	IIIc	52	309	10 months ALC
4	Papillary serous cystadenocarcinoma	IV	58	3187	6 years 1 months ALC
5	Papillary serous cystadenocarcinoma	IIIc	41	NA	8 years 8 months^a^
6	Serous cystadenoma NOS	IIIc	49	463	2 years 7 months^a^
7	Serous cystadenoma NOS	IIIa	64	1404	7 months ALC
Grade 3					
1	Papillary serous cystadenoma	IIIc	75	1443	4 yrs^a^
2	Papillary serous cystadenocarcinoma	IIc	67	24	2 months ALC
3	Serous cystadenocarcinoma NOS	IIIc	55	3025	2 years 5 months until death
4	Serous cystadenocarcinoma NOS	IIIc	59	4224	1 year 4 months until death
5	Serous cystadenocarcinoma NOS	IIIc	76	130	3 years 6 months^a^
6	Serous cystadenocarcinoma NOS	IIc	60	107	3 years 6 months^a^
7	Serous cystadenocarcinoma NOS	IIIc	42	264	10 months until death
8	Serous cystadenocarcinoma NOS	IIIc	58	490	2 years 8 months^a^

*NOS* Not Otherwise Specified, *ALC* At Last Contact

^a^indicates patients were alive at the time of manuscript preparation

**Table 2:** Description of patient tissue samples used for quantitative Real-Time PCR analysis

Sample no.	Diagnosis	FIGO stage	Age	Pre-operative CA125	Survival
Normal					
1	Normal ovarian epithelium	-	NA	-	-
2	Normal ovarian epithelium	-	62	-	-
3	Normal ovarian epithelium	-	45	-	-
4	Normal ovarian epithelium	-	57	-	-
5	Normal ovarian epithelium	-	36	-	-
Grade 3					
1	Serous cystadenocarcinoma NOS	IIIc	55	3025	2 years 5 months until death
2	Serous cystadenocarcinoma NOS	IIc	56	1447	5 years 1 months^a^
3	Papillary serous cystadenocarcinoma	IIIc	75	4831	4 years 1mth until death
4	Serous cystadenocarcinoma NOS	IV	50	7190	1 year 10mth^a^
5	Serous cystadenocarcinoma NOS	IIIc	59	4224	1 year 4 months until death
6	Serous cystadenocarcinoma NOS	IIIc	76	130	3 years 6 months^a^
7	Serous cystadenocarcinoma NOS	IIIc	38	957	2 years 2 months^a^
8	Serous surface papillary carcinoma	IIIc	59	397	4 years 7 months until death
9	Papillary serous cystadenocarinoma	IIIc	64	485	1 year 6 months until death
10	Papillary serous cystadenocarinoma	IIc	68	1934	4 years 9 months^a^
11	Serous cystadenocarcinoma NOS	IIIc	70	459	2 years 5 months until death

*NOS* Not Otherwise Specified

^a^indicates patients were alive at the time of manuscript preparation

To determine whether Oct4A may play a role in the chemoresistant nature exhibited by recurrent EOC tumour cells, we next examined the expression Oct4A in isolated tumour cells derived from the ascites of chemonaïve and recurrent patients (Table 3.3). Oct4A mRNA expression was significantly elevated in tumour cells derived from the ascites of recurrent patients compared to those derived from untreated chemonaive patients (Fig. 1d) (Table 3).

**Table 3:** Description of patient ascites samples used for quantitative Real-Time PCR analysis

Sample no.	Diagnosis	Silverberg grade	FIGO stage	Treatment	Age	Survival
Chemonaive						
1	Serous Cystadenoma/ Early Serous Borderline	NA	NA	None	64	NA
2	Serous Cystadenocarcinoma	NA	III	None	65	1 year 5 months^a^
3	Serous Papillary Carcinoma	G3	NA	None	NA	NA
4	Papillary Serous Cystadenocarcinoma	G3	IIIc	None	48	1 year 6 months^a^
5	Serous Cystadenocarcinoma NOS	G3	IIIa	None	51	1 year 3 months^a^
6	Serous Cystadenocarcinoma NOS	G3	NA	None	71	1 year 1 month^a^
Recurrent						
1	Serous Cystadenocarcinoma NOS	G3	IIc	Carboplatin and Paclitaxel 2 Cycles	64	5 months until death
2	Adenocarcinoma NOS	NA	IV	Carboplatin and Paclitaxel 6 Cycles	67	2 years 8 months until death
3	Serous Cystadenocarcinoma NOS	G3	IIIc	Carboplatin/Paclitaxel/ Bevacizumab-VGEF Inhibitor (ICON7 Trial) 18 Cycles	55	5 years 6 months until death
4	Papillary Serous Cystadenocarcinoma	G3	IIIc	Carboplatin and Paclitaxel 6 Cycles	59	3 years 2 months until death
				Gemicitabine and Cisplatin		
5	Papillary Serous Cystadenocarcinoma	G3	IIIc	Carboplatin/Paclitaxel/BIBF1120- Angiogenesis Inhibitor (OVAR12 Study)	46	3 years 11 months ALC
6	Serous Cystadenocarcinoma NOS	G3	IV	Bevacizumab 1 cycle	57	4 years 3 months
				Paclitaxel and Cisplatin 6 cycles		ALC
				Doxorubicin Pegylated Liposomal 6 cycles		

*NOS* Not Otherwise Specified, *ALC* At Last Contact

^a^indicates patients were alive at the time of manuscript preparation

### Oct4A is over expressed in human ovarian cancer cell lines OVCAR5, SKOV3 and HEY

**Fig. 2:**
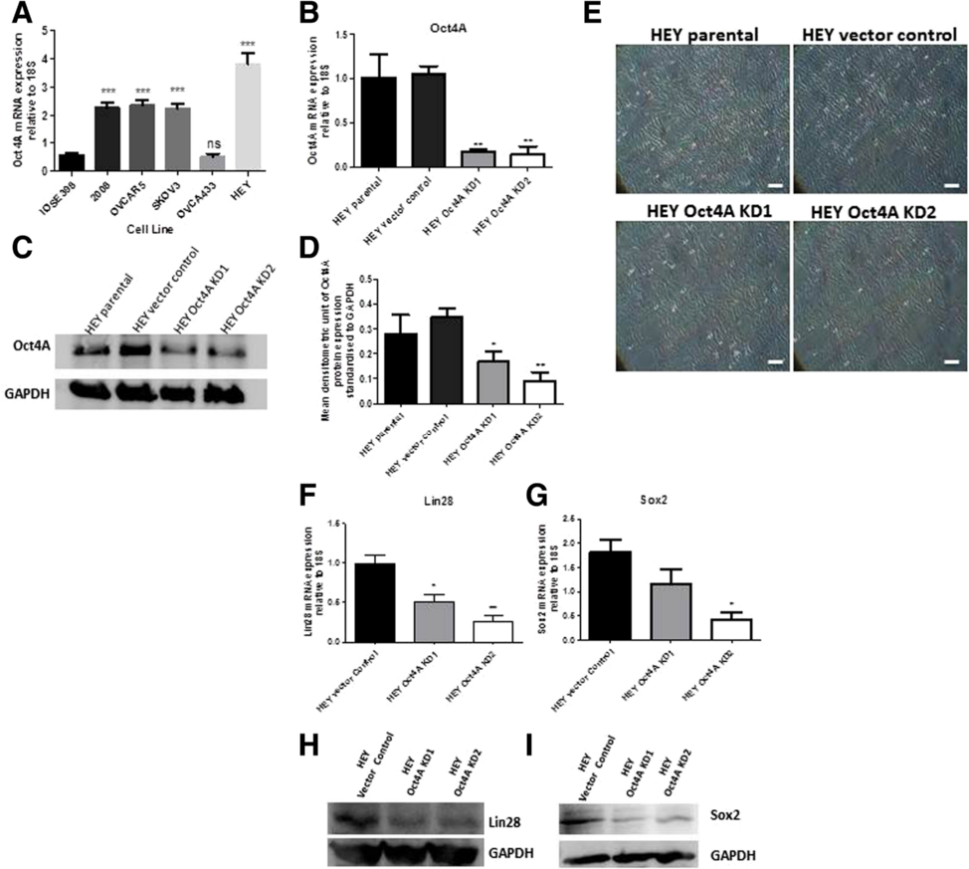
Stable shRNA knockdown of Oct4A in epithelial ovarian cancer cell line HEY. **a** Four established epithelial ovarian cancer cell lines OVCAR5, SKOV3 OVCAR433 and HEY were screened for endogenous expression of Oct4A mRNA by qPCR analysis. Relative quantification of Oct4A mRNA expression was standardized to 18S housekeeping gene and normalized to the normal ovarian epithelium immortalized cell line ISOE398. **b** shRNA-mediated silencing of Oct4A in HEY cells (Oct4A KD1 and Oct4A KD2) was confirmed by qPCR analysis. Relative Oct4A levels were normalized to 18S housekeeping gene and calibrated to vector control samples prepared in triplicate. **c** Western blot analysis using nuclear cell lysates and 12 % SDS-PAGE gels further validating Oct4A knockdown in Oct4A KD cells as determined by protein bands present at ~50 kDa. Total protein load was determined by stripping and re-probing the membrane with GAPDH. **d** Oct4A protein expression was significantly reduced in Oct4A KD cells as determined by densitometry analysis. Data is expressed as a ratio of Oct4A protein expression compared to GAPDH protein expression. **e** Phase contrast images of exponentially growing HEY cells cultured on plastic indicating no obvious morphological changes following shRNA-mediated knockdown of Oct4A. Magnification is set at 100x. Scale bar represents 50 μM. **f**-**g** Lin28 and Sox2 mRNA expression was investigated by qPCR analysis. Relative Lin28 and Sox2 levels were normalized to 18S housekeeping gene and calibrated to vector control samples prepared in triplicate. **h**-**i** Lin28 and Sox2 protein expression in Oct4A KD cells was investigated by Western blot analysis. Cytoplasmic cell lysates were prepared for Lin28 analysis and nuclear cell lysates used for Sox2 analysis. Total protein load was determined by stripping and re-probing the membrane with GAPDH. For all graphs sets, data is expressed as the mean fold change ± SEM from three independent samples. Significance is indicated by *p < 0.05, **p < 0.01 and ***p < 0.001 and determined by One-Way ANOVA using Dunnett's post-test compared to the ISOE398 cell line or HEY vector control

To further examine the expression of Oct4A in EOC, the endogenous expression of Oct4A in the established EOC cell lines OVCAR5, SKOV3, OVCA433 and HEY was investigated by real-time PCR analysis. When compared to normal ovarian surface epithelium cell line ISOE398, the results demonstrated that all cell lines with the exception of OVCA433 displayed significantly increased expression of Oct4A compared to ISOE398 cell line (Fig. 2a). It was also noted, that of all the cell lines, the highly metastatic HEY cell line expressed the greatest endogenous level of Oct4A with an 8-fold increase in Oct4A expression compared to ISOE398 cell line, and an overall 2-fold increase compared to other ovarian cancer cell lines.

### Efficient shRNA mediated knockdown of Oct4A in HEY epithelial ovarian cancer cell line

To determine the potential biological role of Oct4A in ovarian carcinomas, loss of function studies were performed in Oct4A expression abundant HEY cell line using stable shRNA-mediated targeting of Oct4A. HEY cells were transfected with either the shRNA vector control plasmid or the Oct4A targeting shRNA plasmid. Several Oct4A knockdown (KD) clones were produced and evaluated for Oct4A silencing by real-time PCR analysis. Of these, knockdown clones 1&2 (HEY Oct4A KD1 and HEY Oct4A KD2) showed significant suppression of Oct4A mRNA compared to HEY vector control cells with a knockdown efficiency of 80-90 % (Fig. 2b). Oct4A knockdown was seen to be less effective at the protein level with Western blot analysis on nuclear cell lysates suggesting a 40-50 % decrease in Oct4A expression in HEY Oct4A KD1 and Oct4A KD2 cells compared to vector control cells (Figs. 2c & d). As expected there were no differences in the expression of Oct4A mRNA or protein expression in vector control cells when compared to parental cells. Morphologically, the loss of Oct4A expression had no effect on HEY cells (Fig. 2e).

### Knockdown of Oct4A suppressed the expression of Lin28 and Sox2 in monolayer HEY cultures

To investigate whether loss of Oct4A had any effect on the expression of other Oct4-associated genes, we analyzed the expression of embryonic stem cell markers Lin28 and Sox2 in Oct4A KD cells by real-time PCR analysis. The mRNA levels of both Lin28 and Sox2 were suppressed in Oct4A KD cells by >50 % when compared to vector control cells (Figs. 2 f & g). This was further confirmed at the protein level by Western blot analysis (Figs. 2h & i).

### Knockdown of Oct4A altered the spheroid forming ability of HEY cells and suppressed the expression of Lin28 and Sox2 in non-adherent spheroid cultures

**Fig. 3:**
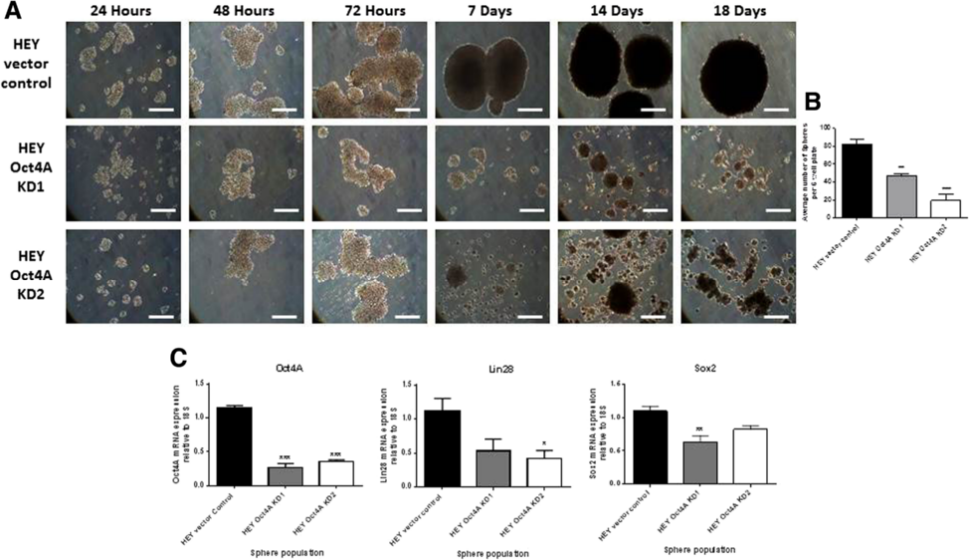
Suppression of Oct4A resulted in reduced spheroid forming ability and a loss of Lin28 and Sox2 in HEY cells. **a** The spheroid forming ability of HEY cells following stable shRNA-mediated knockdown of Oct4A was assessed by culturing cells on ultra-low attachment plates as described in the Materials and Methods. Cellular aggregation was monitored over 18 days and documented using phase contrast microscopy. Images are representative of three independent experiments set at 100x magnification. Scale bar represents 100 μM. **b** The number of spheroids produced after 18 days was assessed using a phase contrast microscope calibrated with an ocular micrometer. Spheroids with a diameter greater than 200 μM were classified as spheroids. **c** Expression of Oct4A, Lin28 and Sox2 in Oct4A KD 18 day spheroids was evaluated by qPCR analysis. Relative quantification of Oct4A, Lin28 and Sox2 mRNA expression in Oct4A knockdown cells was standardized to 18S housekeeping gene and normalized to vector control cells

The ability of tumour cells to form anchorage-independent multicellular aggregates within ascites fluid plays a crucial role in the survival and metastasis of epithelial ovarian tumours [34, 35]. To examine the effects of Oct4A knockdown on the ability of HEY cells to form spheroids, vector control, Oct4A KD1 and Oct4A KD2 cells were cultured on ultra-low attachment plates and monitored over 18 days (Fig. 3a). Vector control cells formed large, compact spheroids with a notable outer rim and were capable of maintaining their integrity within 18 days in culture. In comparison, the ability of both Oct4A KD1 and Oct4A KD2 cells to form spheroids was almost completely abrogated, with both Oct4A KD clones forming smaller and irregular-shaped spheroids which appeared to disintegrate in culture. When assessed at 18 days, the number of spheroids (>200 μM) produced by Oct4A KD1 and Oct4A KD2 cells was significantly reduced (50-80 %) when compared to spheroids produced by vector control cells (Fig. 3b).

Since the formation of multicellular spheroids within ascites fluid has previously been reported to be essential in the preservation and survival of ovarian CSCs [36], the expression of Oct4A, along with Lin28 and Sox2 was investigated in 18 day spheroids produced by vector control and Oct4A KD cells by quantitative real-time PCR analysis (Fig. 3c). Consistent with monolayer cultures, the mRNA expression of Oct4A, Lin28 and Sox2 were significantly reduced in Oct4A KD spheroids (40-70 %) compared to vector control spheroids (Fig. 3c).

### Knockdown of Oct4A reduced the ability of knockdown cell spheroids to adhere to plastic and suppressed the expression of cancer stem cell glycoproteins EpCAM and CD44 in monolayer cultures

**Fig. 4:**
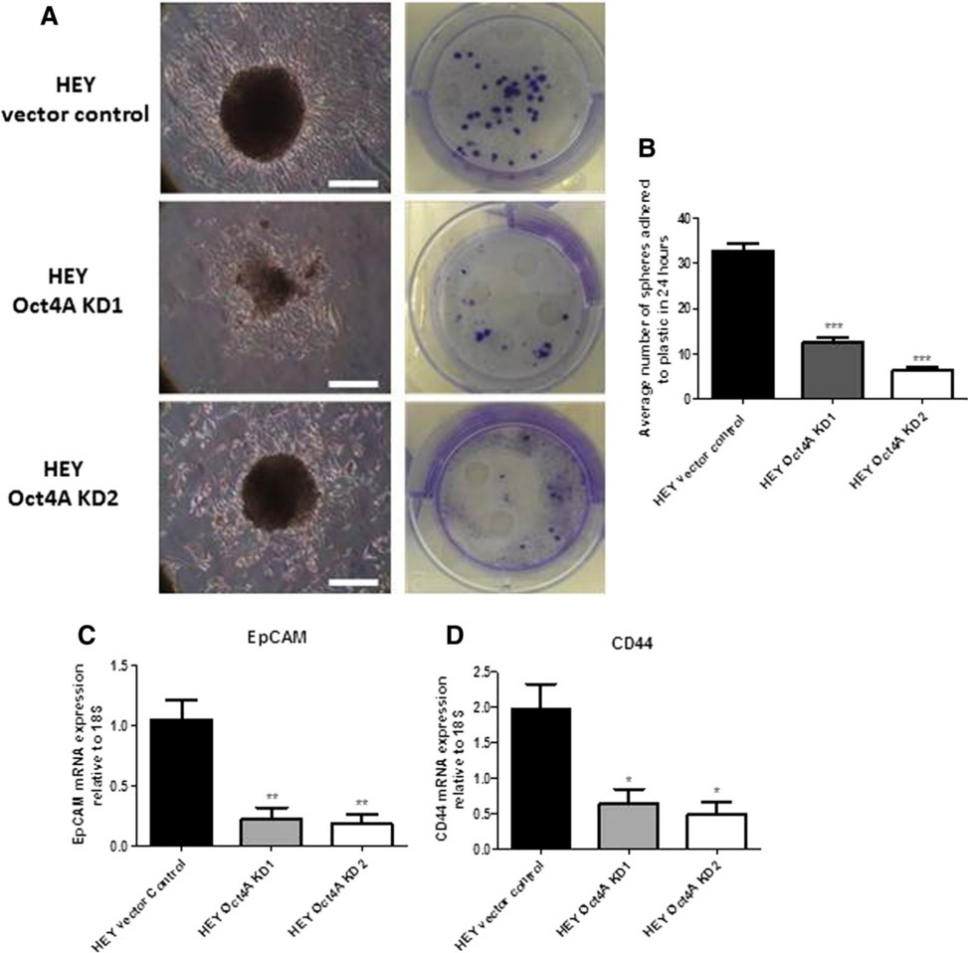
Loss of Oct4A expression resulted in reduced adhesion abilities of spheroids and colony formation in HEY cells. **a** Vector control and Oct4A KD cells were grown as non-adherent spheroids cultures on ultra-low attachment plates as described in the Material and Methods. Following 18 day incubation, spheroids were transferred to 6 well plastic culture plates and incubated for 24 h. Adhered spheroids were then fixed with formalin and stained with 5 % crystal violet. All images are representative of three independent experiments performed in duplicate. Magnification 100x and scale = 100 μM **b** Colony formation in 18 day Oct4A KD spheroids was significantly reduced when compared to vector control spheroids. **c** Expression of adhesion markers CD44 and EpCAM in monolayer Oct4A KD cells was determined by qPCR analysis. Relative quantification CD44 and EpCAM mRNA expression in Oct4A KD cells were standardized to 18S housekeeping gene and normalized to the vector control. All data sets are presented as the mean ± SEM of three independent experiments as determined by One-Way ANOVA and Dunnett's Multiple Comparison post-test compared to vector control. Significance is indicated by *P < 0.05, **p < 0.01 and ***P < 0.001

The ability of ascites tumour spheroids to colonize and invade at distant sites is crucial for the survival and metastasis of EOC tumours [37, 38]. To determine if loss of Oct4A expression in HEY cells reduces the ability of spheroids to adhere to plastic, 24 h colony forming assays were performed on 18 day vector control and Oct4A KD spheroids. Compared to vector control spheroids, a significantly reduced number of Oct4A KD1 and Oct4A KD2 spheroids were capable of adhering to plastic within 24 h (Figs. 4a & b). Furthermore, microscopic analysis of adhered spheroids revealed cells within vector control spheroids were capable of effectively migrating away from the adhered spheroid core. Conversely, cells from adhered spheroids produced by Oct4A KD cells exhibited little to no cellular migration away from the spheroid core (Fig. 4a).

A change in the ability of HEY cells to form tightly compacted spheroids with reduced adhesive abilities suggests alterations in the expression of adhesive proteins may also be effected following loss of Oct4A expression. EpCAM and CD44 are oncogenic glycoproteins commonly expressed by EOC solid tumours and ascites tumour cells [14, 39]. Additionally, both have been associated with a CSC-like phenotype [40, 41]. When compared to vector control cells, a significant loss of EpCAM (>70 %) and CD44 (>60 %) mRNA expression was observed in Oct4A KD monolayer cultures (Fig. 4c).

### Suppression of Oct4A inhibited migratory ability of HEY cells in vitro

**Fig. 5:**
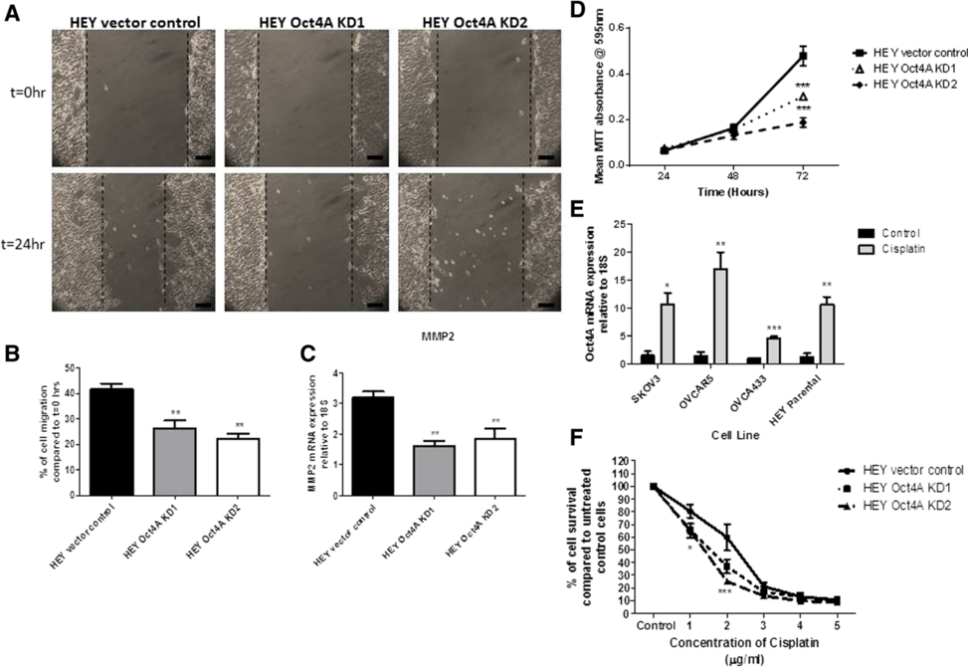
Loss of Oct4A reduced migratory and proliferative abilities in HEY cells while increasing chemosensitivity to cisplatin treatment. **a** The migratory ability of Oct4A KD cells was determined by 24 h wound healing assay. Cells were grown as confluent monolayer prior to the insertion of a single wound and allowed to migrate for 24 h. Images of the wounds were taken at t = 0 h and t = 24 h post wound insertion and are representative of three independent experiments. Images are at 100x magnification and scale bar represents 50 μM. **b** 24 h migration expressed as a percentage of wound closure compared to t = 0 h using an ocular micrometer. Data is presented as the mean ± SEM of three independent experiments. Significance is indicated by **p < 0.01 as determined by One-Way ANOVA compared to vector control. **c** qPCR analysis of MMP2 mRNA expression in Oct4A KD monolayer cells. Relative quantification of MMP2 mRNA expression in Oct4A knockdown cells was standardized to 18S housekeeping gene and normalized the vector control. **d** Proliferative potential of Oct4A KD cells was determined by MTT assay over a 72 h period. Proliferation rates are expressed as the percentage of cell growth compared to 24 h. Data is presented as the mean ± SEM of three individual experiments performed with 6 replicates. Significance at t = 72 h is indicated by *p < 0.05 and ***p < 0.001 as determined by Two-way ANOVA compared to HEY Vector Control. **e** mRNA expression of Oct4A in ovarian cancer cell lines following cisplatin treatment. Relative quantification of Oct4A mRNA expression in ovarian cancer cells was standardized to 18S housekeeping gene and normalized to untreated controls for each cell line. Data is presented as the mean ± SEM of three independent experiments. Significance is indicated by ***p < 0.001, **p < 0.01 and *p < 0.05 compared to the respected untreated control for each cell line as determined by student's test. **f** Loss of Oct4A increased HEY cell sensitivity to 3 day cisplatin treatment as determined by MTT assay. Data is presented as the mean ± SEM of three independent experiments performed in triplicate and expressed as a percentage of cell survival compared to untreated cells for each group. Significance is indicated by *p < 0.05 and ***p < 0.001 for both Oct4A KD1 and Oct4A KD2 at 1 μg/ml and 2 μg/ml as determined by Two-way ANOVA compared to vector control cells

Motility remains an important parameter in tumour metastasis and reflects the ability of tumour cells to migrate away from the original site into neighbouring tissues and distant metastasised sites [42]. To determine whether loss of Oct4A affects the migratory ability of HEY cells, 24 h wound healing assays were performed (Fig. 5a). Suppression of Oct4A in HEY cells had a significant impact on the migration of HEY cells with Oct4A KD cells exhibiting ~50 % reduction in migratory ability compared to vector control cells (Fig. 5b). This was consistent with significantly reduced (~50 %) mRNA expression of MMP2 in Oct4A KD cells compared to vector control cells (Fig. 5c).

### HEY cells exhibited reduced proliferative rates following shRNA-mediated knockdown of Oct4A

Cellular proliferation is fundamental in the survival of tumour cells and in the overall maintenance of a solid tumour mass [43]. Since Oct4A is well known for regulating self-renewal and survival in ESCs [31], the proliferative ability of HEY cells following shRNA-mediated knockdown of Oct4A was investigated using 72 h MTT cell viability assays (Fig. 5d). When compared to vector control cells, both Oct4A KD1 and Oct4A KD2 cells demonstrated significant decrease in cellular proliferation 72 h post plating (30 & 50 % respectively) (Fig. 5d).

### Loss of Oct4A expression enhanced HEY cell sensitivity to cisplatin treatment

As discussed previously, chemotherapeutic resistance has been shown to be attributed by CSCs [13, 15, 44]. To determine whether Oct4A plays a role in chemotherapy induced drug resistance in EOC, the expression of Oct4A was investigated in ovarian cancer cell lines treated with cisplatin for 72 h. Consistent with the profile generated by recurrent chemotherapy treated patient ascites samples, Oct4A mRNA levels were found to be significantly elevated in all cell lines following cisplatin treatment when compared to their untreated control counterparts (Fig. 5e). This elevation suggests a possible role of Oct4A in mediating drug resistance in EOC. The sensitivity of HEY cells to cisplatin following loss of Oct4A was investigated. Compared to cisplatin treated vector control cells, both Oct4A KD1 and Oct4A KD2 cells showed significant increase in sensitivity to 72 h cisplatin treatment as indicated by a decrease in the GI_50_ value (Fig 5f). Overall, vector control cells exhibited a cisplatin GI_50_ value of ~2 μg/ml compared to ~1-1.5 μg/ml for Oct4A KD cells.

### Suppression of Oct4A in HEY cells reduced tumour burden and prolonged survival of mice in xenograft models

**Fig. 6:**
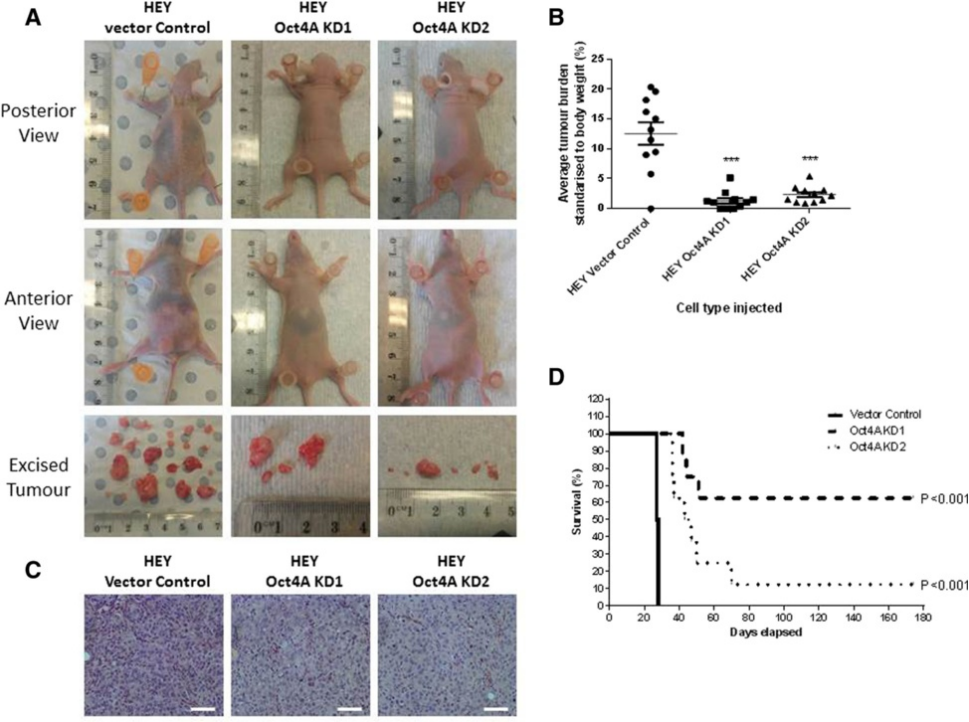
*In vivo* tumour development in mice injected with HEY Oct4A knockdown cells. **a** 5 × 10^6^ vector control, Oct4A KD1 and Oct4A KD2 HEY cells were inoculated by ip injection into 6–8 week old female BALB/c nu/nu mice (*n* = 11/group). 4 weeks post injection tumour development was photographed, mice euthanized and **b** tumour burden calculated. Oct4A KD cells exhibited significantly decreased tumour formation as determined by One Way ANOVA and Turkey’s post-test. Significance is indicated by ***p < 0.001. **c** Excised tumours were subjected to H&E staining. Images are set at 200x magnification and are representative of *n* = 4 per group. Scale bar is set at 50 μM. **d** Prolonged survival status in mice injected with Oct4A KD cells. *n* = 8 mice per Oct4A knockdown group were maintained over a 6 month period and monitored for tumour development. Mice were culled and death reported as mice reached predetermined endpoint criteria (Refer to Methods and Materials for list of criteria). Mice injected with Oct4A KD cells displayed significant increase in mean survival as determined by Kaplan-Meier survival analysis

To further investigate the role of Oct4A in EOC progression *in vivo* intraperitoneal (ip) HEY xenograft mouse models were developed and used as described previously [15]. 4 weeks post inoculation, mice injected with vector control cells displayed several characteristics of advanced stage metastatic disease including abdominal swelling and weight loss (Fig. 6a). Dissection of the abdominal cavity revealed the formation of multiple macroscopic disease deposits primarily visible on the liver, pancreas, large and small bowels. Several smaller tumour nodules were also seeded throughout the entire peritoneal cavity. In comparison, mice injected with either Oct4A KD1 or Oct4A KD2 cells appeared free of ill health and displayed no evidence of tumour formation 4 weeks post inoculation. Only after surgical dissection was it evident that mice injected with Oct4A KD cells produced significantly smaller tumours and an overall reduced tumour burden (Figs. 6a & b). Despite obvious growth reductions, histological examination of excised xenograft tumours revealed that Oct4A KD cells-derived tumours retained similar morphology to that produced by vector control cells (Fig. 6a). Further microscopic investigation revealed however, that tumours derived from Oct4A KD cells exhibited a higher cytoplasm/nucleus ratio compared to those produced by HEY vector control cells (Fig. 6c).

Since, the majority of mice injected with Oct4A KD cells displayed little to no tumour development at 4 weeks, the survival rates of mice injected with Oct4A KD1 and KD2 cells was assessed over a 6 months period (Fig. 6d). Within 28 days however, 100 % of mice inoculated with vector control cells developed debilitating tumours and required euthanization. In contrast, mice injected with Oct4A KD1 cells displayed an average survival period of 125.8 days (4.5-fold increase compared to vector control mice), while those injected with Oct4A KD2 cells exhibited a survival period of 61.6 days (2.2-fold increase). In total, 60 % (5/8) of mice injected with HEY Oct4A KD1 cells and 12.5 % (1/8) of mice injected with HEY Oct4A KD1 cells failed to produce any evidence of tumour formation over the 6 month analysis period (experiment endpoint). These results suggest that suppression of Oct4A not only reduces tumour burden in mice but significantly delays the onset of tumour formation.

### Oct4A suppression in HEY cells abrogated the invasive ability of tumour cells to vital organs in in vivo mouse models

**Fig. 7:**
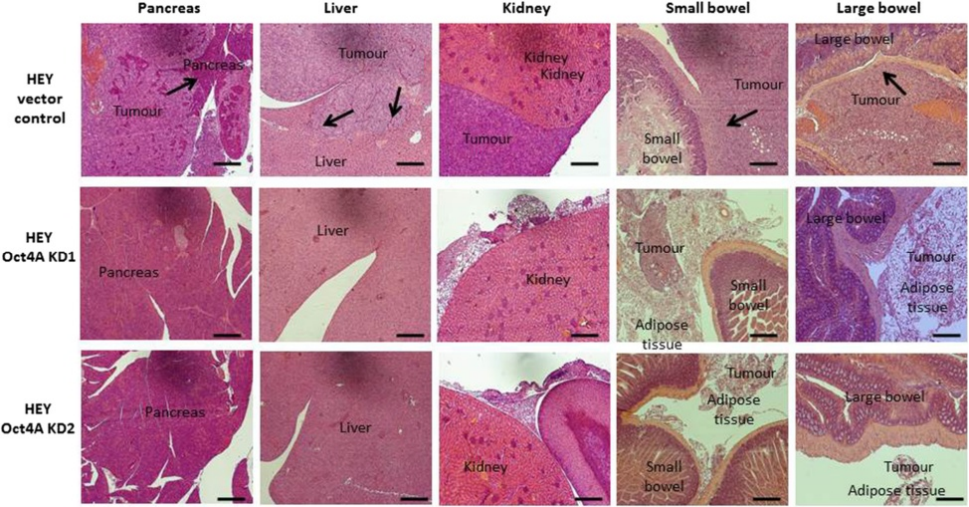
H&E staining of organ infiltration by vector control, Oct4A KD1 and OCT4A KD2 HEY cells. Representative H&E images of pancreas, liver, kidney, small and large bowels in mice injected with HEY vector control, Oct4A KD1 and Oct4A KD2 cells (*n* = 4/mouse group). Images show vector control cells infiltrating all organs with the exception of the kidneys. Oct4A KD cells do not undergo organ infiltration with tumour deposits only found within sections of adipose tissue. Arrows indicate tumour cells invading respective organs. Magnification is set at 100x. Scale bar represents 100 μM

To further evaluate the role of Oct4A in EOC tumour metastasis, tumour organ infiltration patterns generated by vector control and Oct4A KD cells 4 weeks post inoculation were assessed using H&E staining. In line with our previous studies [15], mice injected with vector control cells produced tumours which infiltrated the pancreas, liver, kidney, small and large bowels (Fig. 7 & Additional file 2: Figure S2). While, most of the metastases located on the lungs, kidneys and bowels were seen to focally penetrate the respective organ, those located on the liver were seen to penetrate deep within the organ. Remarkably, vector control tumour cells invading the pancreas were seen to completely replace the organ. On the other hand, mice injected with Oct4A KD cells displayed no evidence of organ infiltration by tumour cells, with tumour deposits found only in areas of adipose tissue surrounding the kidney, small and large bowels.

### Knockdown of Oct4A significantly reduced the expression of Oct4, Sox2, CA125, Ki67 and Bcl2 in xenografts derived from Oct4A KD cells compared to vector control xenografts

**Fig. 8:**
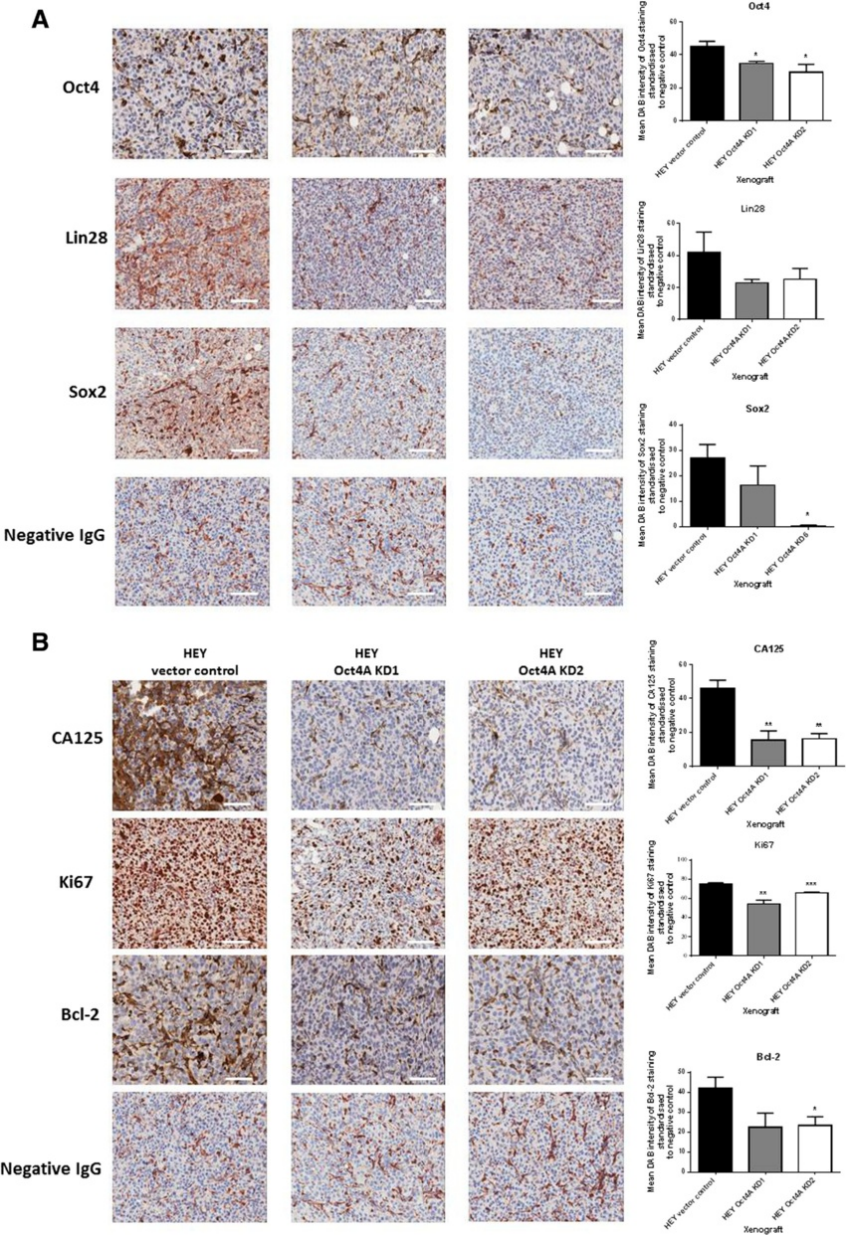
**a** Expression of Oct4, Lin28, Sox2, CA125, Ki67 and Bcl-2 in mouse tumour xenografts generated by vector control, Oct4A KD1 and Oct4A KD2 cells. Representative immunohistochemistry staining images of debulked mouse xenografts for the expression of Oct4, Lin28 and Sox2. **b** Representative immunohistochemistry staining images of debulked mouse xenografts for the expression of CA125, Ki67 and Bcl-2. All images are set at 200x magnification and scale bar represents 50 μM. Quantification of antibody staining was determined by using Image J software recognizing DAB intensity. Variations in staining were determined by subtracting the negative control DAB reading from the protein of interest DAB reading for each xenograft. Data is presented as the mean ± SEM of staining intensity (n = 4/group). Significant variations between Oct4A KD groups and vector control were determined by student’s t-test *p < 0.05; **p < 0.01

Immunohistochemistry analysis was performed on surgically excised tumours from mice inoculated with vector control and Oct4A KD cells. Consistent with monolayer and spheroid cultures, the expression of Oct4 and Sox2 was significantly reduced in tumours derived from Oct4A KD cells compared to those derived from vector control cells (Fig. 8a). The expression of Lin28 was also markedly reduced in Oct4A KD tumour xenografts, however this was not statistically significant. Additionally, the expression of Ki67 and Bcl-2 were significantly reduced in Oct4A KD xenografts and was consistent with overall reduced tumour size in Oct4A KD xenografts (Fig. 8b). Immunostaining also revealed that CA125 expression was significantly reduced in Oct4A KD xenograft tumours compared to those derived from vector control cells. This suggests that the overall tumour forming ability of HEY cells *in vivo* has been suppressed following loss of Oct4A expression *in vitro*.

## Discussion

Despite extensive treatment regimes, ascites-mediated recurrence continues to remain a critical process in the progression of EOC [36]. Understanding the molecular mechanisms which contribute to the survival of exfoliated primary ovarian tumour cells in the peritoneal cavity therefore remains vital in the management of the disease. During the past few years, the embryonic transcription factor Oct4 has received considerable attention in cancer stem cell biology and its expression has been reported in a wide range of tumours [45]. However, unfamiliarity with the several known Oct4 isoforms, as well as the existence of a high number of pseudogenes [30] has led to the misinterpretation of several results surrounding Oct4A in the context of stem cell biology [30]. A more rigorous analysis of the specific Oct4 isoforms is therefore required to elucidate their functional roles in cancer.

Although the expression of Oct4 has previously been reported in ovarian tumours [46] and patient ascites samples [47], this is the first study to our knowledge to demonstrate the expression of the specific Oct4A isoform in serous ovarian tumours and chemotherapy-treated recurrent serous patient ascites samples. We demonstrate that Oct4A is significantly elevated in serous borderline tumours compared to normal ovarian tissues and that this elevated expression is retained throughout the histological spectrum of serous ovarian tumours. Due to a small sample size used in the current study, no elucidation between the elevated expression of Oct4A and patient’s prognosis could be determined. However, this combined with the significant elevation of Oct4A expression in the tumour cells isolated from the ascites of recurrent patients compared to those from chemonaive patients suggests that elevated expression of Oct4A may not only have a role in ovarian cancer progression but may also facilitate recurrence. Interestingly and in this context, the Oct4A-abundant HEY cell line has previously been shown to form highly robust and aggressive tumours in mouse models [15]. Alternatively, the Oct4A low expressing OVCA433 is not tumourigenic in *in vivo* mouse models [48]. Such observations may potentially correlate to the proposed CSC-like functional role of Oct4A in regulating ovarian tumour growth and survival [35, 49].

The current literature on the functional role of Oct4 in epithelial ovarian cancer is relatively sparse, with the transcription factor primarily being used as a marker to detect CSC-like cells in primary ovarian tumours and patient ascites samples [50]. In this study using shRNA-mediated knockdown of Oct4A, we provide evidence to support the concept that the Oct4A isoform may be a key regulatory factor associated with cancer stem cell-like properties in serous ovarian tumours. It has previously been shown that cancer cells cultured as spheroids exhibit enhanced tumourigenic and metastatic ability combined with increased expression of CSC-like genes [36]. Here we demonstrate that knockdown of Oct4A in HEY ovarian cancer cell line not only significantly diminishes the anchorage-independent growth of ovarian cancer cells as spheroids, but results in the decreased expression of oncogenic and CSC-like Lin28 and Sox-2 expressions compared to vector control cells. Since both Lin28 and Sox2 function to maintain self-renewal in pluripotent stem cell populations [51, 52], this result further emphasizes the role of Oct4A as a master regulator of self-renewal in stem cell-like populations. It is also the first study to suggest the regulation of Lin28 and Sox2 by the specific Oct4A splice variant in EOC. The expression of both EpCAM and CD44 were also significantly reduced in knockdown cells compared to vector control cells, suggesting that the loss of anchorage independent state as spheroids in Oct4A knockdown cells may be due to the loss of cell-cell adhesive molecules such as EpCAM and CD44. The interaction between CD44 and Oct4 has previously been described in squamous cell tumours [53], and EpCAM and CD44 have previously been implicated in malignant ovarian tumours [54, 55]. However, the association between EpCAM and CD44 with regards to the specific Oct4A isoform remains to be identified. We also demonstrate that loss of Oct4A expression resulted in a significant reduction in proliferation and migration of HEY cells, emphasizing the vital role of Oct4A in tumour cell survival and migration. Loss of Oct4A expression also correlated with significantly reduced expression of MMP2 suggesting that the invasive ability of HEY cells has been reduced following Oct4A knockdown. These observations were further validated in mouse xenograft experiments which showed significantly reduced tumour burden, tumour organ invasion and overall significantly enhanced survival of mice injected with Oct4A knockdown cells compared to vector control cells. In addition, tumour xenografts derived from Oct4A knockdown cells displayed relatively lower abundance of markers associated with ovarian cancer including oncogenic markers Lin28 and Sox2, along with proliferation maker Ki67 and anti-apoptotic Bcl-2 expression. This indicates that reduced tumourigenic ability, combined with a reduction in proliferative ability mediated by a loss of Oct4A may have slowed or abrogated tumour formation and growth. Interestingly, tumour xenografts generated from Oct4A knockdown HEY cells exhibited significantly lower CA125 expression. Elevated levels of CA125 are a hallmark characteristic in ovarian cancer diagnosis and is frequently observed in relapsed ovarian cancer patients [56] and suggests that the tumours generated by Oct4A knockdown cells are overall less tumourigenic and aggressive compared to those with abundant Oct4A expression.

Overexpression of Oct4 has previously been implicated with chemoresistance in tumours [57]. Here we demonstrate significant elevation of Oct4A expression in established EOC cells which survived cisplatin treatment. This correlated with significantly elevated expression of Oct4A in the isolated tumour cells derived from the ascites of chemoresistant recurrent EOC patients compared to those of chemonaive patients. This highlights the potential role that Oct4A may have in the resistant phenotype currently exhibited by tumour cells of EOC patients. Interestingly, this observation was further reinforced after loss of Oct4A expression in HEY cells which resulted in the increase in the sensitivity to cisplatin treatment, suggesting a direct role of Oct4A in cisplatin-mediated drug resistance which is critical for ovarian tumour recurrence.

## Conclusions

**Fig. 9:**
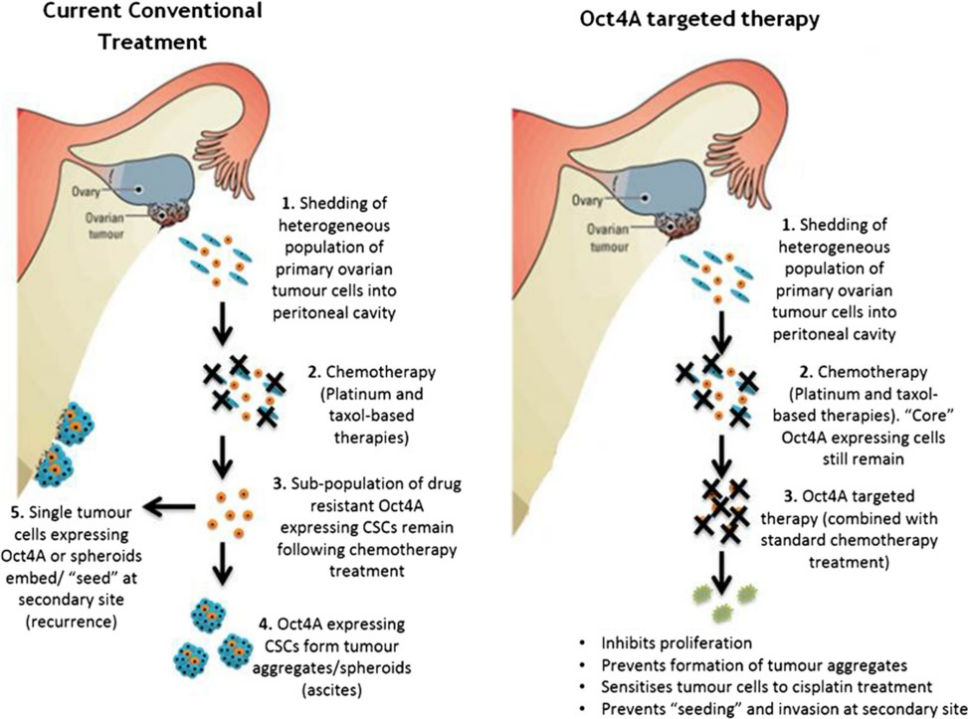
A proposed model of Oct4A-mediated epithelial ovarian recurrence: **a** Current model of Oct4A mediated recurrence driven by drug resistant Oct4A-expressing EOC ascites tumour cells. A population of Oct4A-expressing tumour cells which disseminate directly into the peritoneal cavity are capable of surviving traditional combination therapy consisting of cisplatin and paclitaxel. These cells maintain long term tumourigenicity by the formation of multicellular tumour aggregates (spheroids) or directly 'seed' at a metastatic site to initiate secondary/recurrent disease. **b** Model of Oct4A targeted therapy. Oct4A-expressing primary ovarian tumour cells would still be capable of exfoliating directly into the peritoneal cavity. In contrast however, Oct4A expressing ascites tumour cells would be targeted by Oct4A-specific therapy (potentially in combination with cisplatin and paclitaxel treatment) resulting in the inhibition of tumour cell survival and the prevention of ongoing cancer progression/recurrence

In summary, this study highlights new insights into the biology of Oct4A in serous ovarian cancer and indicates that Oct4A plays a crucial role in serous ovarian tumor progression, survival, chemoresistance and metastasis. It could therefore be hypothesised that following exfoliation into the peritoneal cavity, Oct4A expressing primary EOC tumour cells are capable of long term survival through ongoing self-renewal, tumourigenicity and chemoresistance which would contribute to the recurrence of EOC tumours (Fig. 9). We conclude that enhanced expression of Oct4A in the ascites-derived tumour cells can be potentially used as an indicator of chemoresistance and subsequent for relapse in serous ovarian cancer patients. Targeting Oct4A through novel therapeutics may provide an important strategy to overcome ovarian cancer metastasis and chemoresistance.

## Materials and methods

### Patient samples

#### Tissue collection

Primary serous epithelial ovarian tumours and normal ovarian tissues were obtained from patients requiring surgical resection after obtaining written informed consent under protocols approved by the Human Research and Ethics Committee (HREC approval # 09/09) of The Royal Women’s Hospital, Melbourne, Australia. The histopathological diagnosis, tumour grades and stages were determined by anatomical pathologists at the Royal Women’s Hospital as part of clinical diagnosis (Tables 1&2). Patients who were treated with chemotherapy prior to surgery were excluded from specimen collection. Tissues were paraffin embedded or snap frozen at the time of collection and stored at −80 °C until processed.

#### Ascites collection

Ascites was collected from patients diagnosed with Stages III-IV ovarian serous cystadenocarcinoma or adenocarcinoma Not Otherwise Specified (NOS) after obtaining written informed consent under the protocols approved by the HREC approval #09/09. The histopathological diagnosis, tumour grades and stages were determined by anatomical pathologists at the Royal Women’s Hospital as part of clinical diagnosis (Table 3). Ascites collected from patients at the time of diagnosis and prior to the commencement of treatment were termed chemonaïve (CN). Ascites were also collected from patients at the time of recurrence (CR). Patients in this group had previously received different chemotherapy combinations as described in Table 3. All patients have been classified according to their survival status as either 'alive' or 'deceased' at the time of manuscript preparation. Those labeled as 'At Last Contact (ALC)' were no longer receiving treatment at The Royal Women's Hospital at the time of manuscript preparation. Consequently, their current status remains unknown.

### Preparation of tumour cells from ascites of ovarian cancer patients

Tumour cells from ascites were isolated as previously described [14].

### Cell lines and media

Four established human epithelial ovarian cancer cell lines SKOV3, OVCAR5, OVCA433, and HEY were used in this study. The growth conditions of these cell lines have been described previously [58]. The human ovarian surface epithelial cell line (IOSE398) transfected with the SV-40 antigen was obtained from Dr Nelly Auersperg, University of British Columbia, Canada [59]. Cells were routinely checked for mycoplasma infection.

### Antibodies

Monoclonal antibody against human Oct4A and the secondary anti-mouse IgG HRP antibody was obtained from R&D Systems (Minneapolis, Minnesota, USA). Monoclonal and polyclonal antibodies against human Sox2 and Lin28 were obtained from Cell Signalling Technology (Danvers, Massachusetts, USA). The polyclonal antibody against GAPDH was obtained from IMGENIX (San Diego, CA, USA). Secondary anti-rabbit IgG antibody was obtained from Millipore (Billerica, MA, USA). DAPI Nucleic acid stain and Alex Fluor® 488 goat anti-mouse IgG were obtained from Life Technologies (Carlsbad, CA, USA).

### Immunohistochemistry

Immunohistostaining of primary human tissue specimens for Oct4A was performed using components from the CINtek® p16 Histology Kit (Roche) as described by the manufacturer. Sections were assessed microscopically for positive DAB staining and quantified using the open source image processing package Fiji (Fiji Is Just ImageJ) with a plug-in developed to recognize total DAB staining. Refer to the Supplementary Method for a detailed methodology.

### RNA extraction and real-time PCR

Quantitative real-time PCR was performed as described previously [14]. Relative quantification of gene expression was normalized to 18S and calibrated to the appropriate control sample using the SYBR Green-based comparative CT method (2^-ΔΔCt^). The primer sets for Oct4A Lin28, Sox2, EpCAM and CD44 are described in Table 4. The probe for 18S has been described previously [46].

**Table 4:** Primer sequences of oligos used in quantitative Real-Time PCR

Oligo name	Forward (F) 5’-3’	Primer sequence (5'-3')
	Reverse (R) 5’-3’	
Oct4A	F	CTC CTG GAG GGC CAG GAAT C
	R	CCA CAT CGG CCTG TGT ATA T
Lin28	F	CAA AAG GAA AGA GCA TGC AGA AG
	R	GCA TGA TGA TCT AGA CCT CCA CA
Sox2	F	ATG CAC CGC TAC GAC GTG A
	R	CTT TTG CAC CCC TCC CAT TT
CD44	F	CCA ATG CCT TTG ATG GAC CA
	R	TGT GAG TGT CCA TCT GAT TC
EpCAM	F	CGT CAA TGC CAG TGT ACT TCA GTT G
	R	TCC AGT AGG TTC TCA CTC GCT CAG

### shRNA transfection of HEY ovarian cancer cell line

The HEY-Oct4A shRNA clones (Oct4A KD1 and Oct4A KD2) were generated by transfecting cells with the pLKO.1 Mission shRNA custom DNA construct (Sigma-Aldrich) using FuGENE HD transfection reagent (Roche) following the manufacturer’s protocol and using Puromyocin selection. The target sequence for the Oct4A construct was based on published genomic sequences of Oct4A [31] on human chromosome 6 (accession no. NM_002701) and retrieved from the public databases at the National Center for Biotechnology Information (www.ncbi.nlm.nih.gov). The target sequence is as follows: CCTTCGCAAGCCCTCATTTCA. Vector control HEY cells were generated by stably transfecting a non-target shRNA negative control.

### Western blotting

Cell lysates were extracted using the NU-PER nuclear and cytoplasmic extraction kit (Thermo Scientific, Waltham, MA, USA) as per manufacturer's instructions. SDS-PAGE and Western blot was performed on the cell lysates as described previously [13]. All membranes were probed for 48 h with primary Oct4A, Lin28 or Sox2 antibodies set at 1:1000 dilutions.

### Spheroid forming assay

The spheroid forming ability of cells was determined by seeding cells in ultra-low attachment 6 well culture plates as described previously [13]. Cellular aggregates with a diameter greater than 200 μm were classified as spheroids.

### Spheroid viability assay

The viability of 18 day spheroids was assessed by directly seeding spheroids onto 6 well plastic culture plates and culturing for 24 h at 37 °C in the presence of 5 % CO_2_. Adherent spheroids were formalin fixed, stained with 5 % crystal violet and counted manually.

### Cell migration assays

The migratory ability of cells was assessed by wound healing assay as described previously [13]. Quantitative analysis of cell migration was determined by measuring the width of the wound at 10 randomly selected points at t = 0 h and t = 24 h post wound incision using an ocular micrometer. Migration was expressed as a percentage of 24 h wound closure compared to 0 h.

### Proliferation assays

3-(4, 5-dimethylthiazol-2-yl)-2, 5-diphenyl-tetrazolium bromide (MTT) (Sigma-Aldrich) assays were used to quantify cell proliferation rates as described previously [45]. Briefly, 4 x 10^3^ cells were seeded in 96-well plates and cultured at 37 °C in the presence of 5 % CO_2_ until required for analysis at 24 h, 48 h and 72 h time points. On the day of analysis, the growth media was discarded and MTT (dissolved in 1X PBS solution; final concentration 0.5 mg/mL in 1 X PBS) was added. Cellular metabolism of MTT was permitted to occur at 37^0^ in the presence of 5 % CO_2_. Following incubation, the MTT reagent was removed, 100 μL of dimethyl sulfoxide (DMSO) added and cells left to incubate for 10 mins in to dissolve formazan crystals. Samples were read at OD_595nm_ using the SpectraMax190 Absorbance Microplate Reader and SoftMax® Pro Computer Software (Molecular Devices). Proliferation rates are expressed as a percentage of cell growth compared to 24 h.

### Chemosensitivity assays

The 50 % growth inhibition (GI_50_) value for cells in response to 72 h cisplatin treatment was performed using MTT assays as described previously [45]. Briefly, 4 X 10^4^ cells were seeded in 96-well plates in appropriate growth medium and incubated for 24 h in humidified atmosphere at 37 °C in the presence of 5 % CO_2_. Following 24 h incubation, culture media was removed and substituted with complete growth media containing increasing concentrations of cisplatin. Cells were subjected to 72 h cisplatin treatment and the chemosensitivty of each cell line determined by standard MTT assay as described above. Chemosensitivity is expressed as a percentage of cell survival at 72 h compared to untreated control cells.

### Animal studies

#### Animal ethics statement

This study was carried out in strict accordance with the recommendations in the Guide for the Care and Use of the Laboratory Animals of the National Health and Medical Research Council of Australia. The experimental protocol was approved by the Department of Surgery, Royal Melbourne Hospital, Australia Animal Ethics Committee (Project-006/11).

#### Animal experiments

Animal experiments were performed as described previously [15]. Metastatic development was documented by a Royal Women's Hospital pathologist according to histological examination (H&E staining) of the organs as described previously [20].

#### Immunohistochemistry of mouse tumours

Immunohistochemistry analysis of mouse tumours was performed as described previously [20]. Primary antibodies against human Oct4, Lin28, Sox2, Ki67, Bcl-2 and CA-125 were diluted according to the manufacturer's instruction. Primary antibody staining was detected using the ultraView Universal DAB detection kit (Roche). Negative controls were prepared for each section throughout the staining process by incubating tumour xenografts in the absence of primary antibodies. Images of immunohistochemical staining were taken using an Aperio ImageScope (Leica Microsystems, Mt Waverly, Australia) and associated digital pathology viewing software. DAB staining was measured by taking a minimum of 10 images per tumour section and running the images through the open source image processing package Fiji (Fiji Is Just ImageJ) with a plug-in developed to recognise DAB staining.

### Statistical analysis

All results are presented as the mean ± standard error of the mean (SEM) of three independent experiments. Statistical significance was measured compared to the vector control using one way-ANOVA and Dunnett’s Multiple Comparison test unless otherwise indicated. A probability level of <0.05 was adopted throughout to determine statistical significance.

## Additional files

Additional file 1: Figure S1. Evidence of Oct4A expression and localization in additional primary serous epithelial ovarian tumour samples. Immunohistochemical staining of Oct4A in normal ovary, borderline, grade 2 and grade 3 primary ovarian tumours. Positive Oct4A expression is indicated by intense nuclear staining. Images are set at 200x. Scale bars represent 10 μM. (JPEG 58 kb) Additional file 2: Figure S2. H&E staining of organ infiltration by vector control, Oct4A KD1 and OCT4A KD2 HEY cells (200x magnification). Representative H&E images of pancreas, liver, kidney, small and large bowels in mice injected with vector control, Oct4A KD1 and Oct4A KD2 cells (*n* = 4/mouse group). Images show vector control cells infiltrating all organs with the exception of the kidneys. Oct4A KD cells do not undergo organ infiltration with tumour deposits only found within sections of adipose tissue. Arrows indicate tumour cells invading respective organs. Magnification is set at 200x. Scale bar represents 100 μM. (JPEG 80 kb)
