# Supplementary figures and images for: A critical role of Oct4A in mediating metastasis and disease-free survival in a mouse model of ovarian cancer
Source: Mol Cancer. 2015 Aug 11;14:152. doi: 10.1186/s12943-015-0417-y (PMC4531496; doi:10.1186/s12943-015-0417-y)

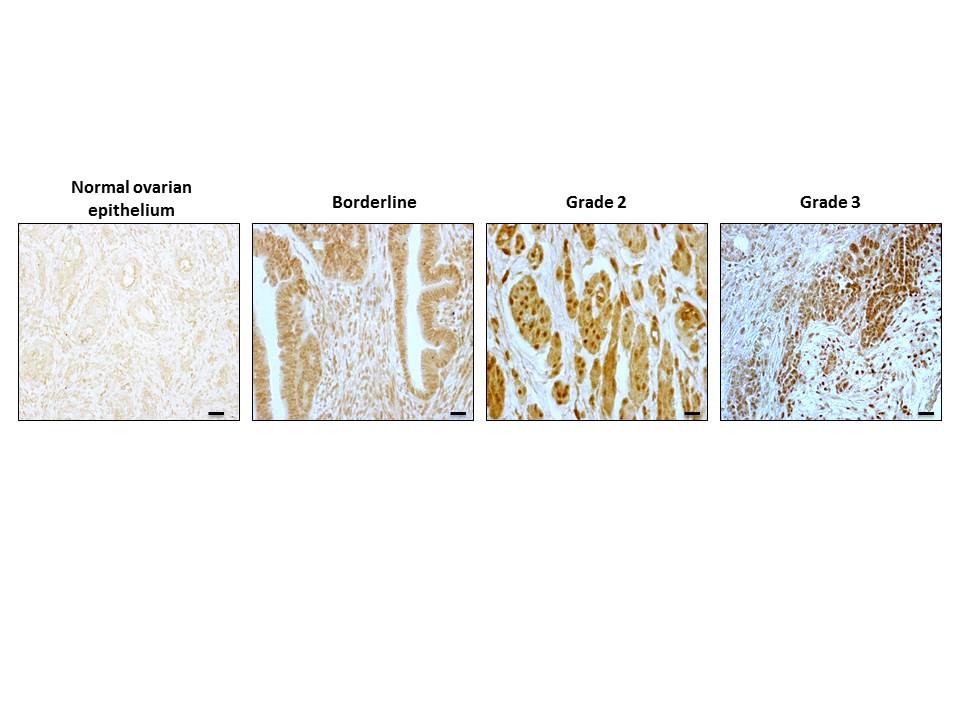

Supplement: Additional file 1: Figure S1. — Evidence of Oct4A expression and localization in additional primary serous epithelial ovarian tumour samples. Immunohistochemical staining of Oct4A in normal ovary, borderline, grade 2 and grade 3 primary ovarian tumours. Positive Oct4A expression is indicated by intense nuclear staining. Images are set at 200x. Scale bars represent 10 μM. (JPEG 58 kb) [file 12943_2015_417_MOESM1_ESM.jpg]

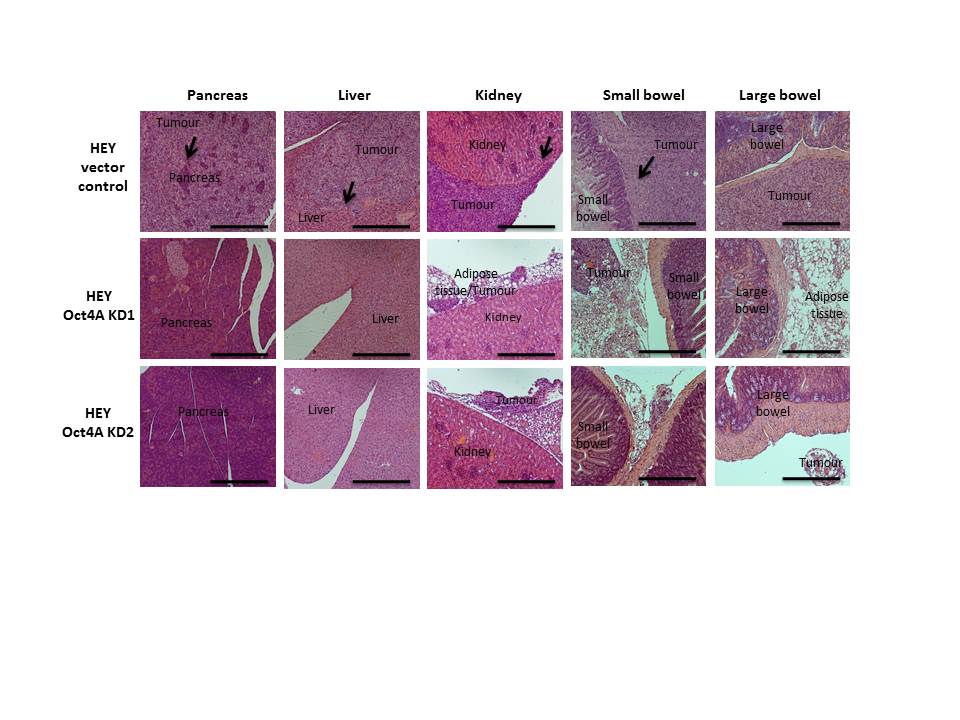

Supplement: Additional file 2: Figure S2. — H&E staining of organ infiltration by vector control, Oct4A KD1 and OCT4A KD2 HEY cells (200x magnification). Representative H&E images of pancreas, liver, kidney, small and large bowels in mice injected with vector control, Oct4A KD1 and Oct4A KD2 cells (n = 4/mouse group). Images show vector control cells infiltrating all organs with the exception of the kidneys. Oct4A KD cells do not undergo organ infiltration with tumour deposits only found within sections of adipose tissue. Arrows indicate tumour cells invading respective organs. Magnification is set at 200x. Scale bar represents 100 μM. (JPEG 80 kb) [file 12943_2015_417_MOESM2_ESM.jpg]
